# Alzheimer’s Disease and Oral Health from Clinical Challenges to Interdisciplinary Care: A Narrative Review

**DOI:** 10.3390/jcm14196696

**Published:** 2025-09-23

**Authors:** Diana Tatarciuc, Florin Razvan Curca, Dragos Ioan Virvescu, Oana Maria Butnaru, Ancuta Goriuc, Simona Bida, Ionut Luchian, Zinovia Surlari, Mihaela Scurtu, Ramona Gabriela Ursu, Dana Gabriela Budala

**Affiliations:** 1 Department of Internal Medicine, Faculty of Medicine, “Grigore T. Popa” University of Medicine and Pharmacy, 700115 Iasi, Romania; 2 Department of Partial Dentures and Implantology, Faculty of Dental Medicine, “Grigore T. Popa” University of Medicine and Pharmacy, 700115 Iasi, Romania; 3 Department of Dental Materials, Faculty of Dental Medicine, “Grigore T. Popa” University of Medicine and Pharmacy, 700115 Iasi, Romania; 4 Department of Biophysics, Faculty of Dental Medicine, “Grigore T. Popa” University of Medicine and Pharmacy, 700115 Iasi, Romania; 5 Department of Biochemistry, Faculty of Dental Medicine, “Grigore T. Popa” University of Medicine and Pharmacy, 700115 Iasi, Romania; 6 Socola Institute of Psychiatry, Faculty of Medicine, “Grigore T. Popa” University of Medicine and Pharmacy, 700115 Iasi, Romania; 7 Department of Periodontology, Faculty of Dental Medicine, “Grigore T. Popa” University of Medicine and Pharmacy, 700115 Iasi, Romania; 8 Department of Fixed Prosthodontics, Faculty of Dental Medicine, “Grigore T. Popa” University of Medicine and Pharmacy, 700115 Iasi, Romania; 9 Department of Microbiology, Faculty of Medicine, “Grigore T. Popa” University of Medicine and Pharmacy, 700115 Iasi, Romania; 10 Department of Complete Dentures, Faculty of Dental Medicine, “Grigore T. Popa” University of Medicine and Pharmacy, 700115 Iasi, Romania

**Keywords:** Alzheimer’s disease, oral health, cognitive decline, dementia, periodontal disease

## Abstract

The link between oral health and Alzheimer’s disease (AD) has gained increasing attention in recent years. Emerging evidence suggests that this association is bidirectional, involving both biological mechanisms and behavioral consequences that reinforce one another over time. **Literature Review**: A narrative synthesis of systematic reviews, meta-analyses, and scoping reviews published between January 2010 and March 2024 was conducted. Searching was performed in four electronic databases (PubMed, Scopus, the Web of Science, and the Cochrane Library), using a combination of MeSH terms and free-text keywords related to dementia and oral health. Inclusion criteria targeted human studies published in English with full-text access and a clear focus on the interplay between oral status and Alzheimer’s disease. **Results**: The reviewed literature indicates that periodontal disease, tooth loss, and oral microbiome alterations may contribute to neuroinflammation and cognitive decline, potentially influencing the onset and progression of AD. Conversely, Alzheimer’s disease negatively affects oral health through impaired self-care, reduced motor coordination, salivary changes, and altered pain perception. **Conclusions**: By mapping out these interconnections, the findings support a shift in perspective; oral health should be considered a relevant factor in both the prevention and management of Alzheimer’s disease. Dentistry and neurology must move closer together in clinical practice, particularly in the care of older adults. Promoting oral health is not just about preserving teeth; it may be part of preserving cognitive function and quality of life.

## 1. Introduction

The link between oral health and cognitive performance is being studied more, underlining the link between brain health and systemic well-being [1]. This changing view emphasizes that dental health, especially in senior people with neurological diseases like Alzheimer’s, affects overall health [2].

Alzheimer’s disease (AD), the leading cause of dementia, affects over 50 million people globally, and this is expected to quadruple by 2050 [3]. This neurological disease mostly affects those over 65.

AD causes cognitive deterioration, behavioral changes, dependence, and high healthcare costs. Neuropathological hallmarks include amyloid-β accumulation, tau protein hyperphosphorylation, synaptic dysfunction, and neuronal death. Despite its genetic, vascular, and inflammatory causes, there is no cure [4]. These elements emphasize the necessity of studying modifiable risk variables, including dental health, that may affect disease development and progression [5]. Many factors enhance the risk of AD, including age, gender, inheritance, co-morbidities like metabolic and cardiovascular illnesses [6], and environmental factors [7,8].

Alzheimer’s disease (AD) causes cognitive decline that progresses from daily challenges to dependency and death [9]. Neuropathologically, AD causes neuronal death and brain atrophy due to amyloid-β (Aβ) plaque accumulation and neurofibrillary degeneration [10]. Extensive study has failed to determine its cause, and no cure or prevention is available. Lack of treatment and rising prevalence make AD a substantial healthcare burden and highlight the need to understand its processes [10].

Researchers are investigating many causes of AD, including oral health. Oral diseases such periodontitis, caries, and fungal infections can spread throughout the body [11]. Several studies suggest a complex relationship between dental health and AD progression, notably the oral microbiota [12]. Oral microbiota in the brain may contribute to AD formation and progression through neuroinflammation, tau phosphorylation, and amyloid-beta buildup [13]. However, cognitive and functional impairment in AD patients makes it difficult to maintain good dental hygiene, creating a cycle of poor oral and cognitive health [14].

Recent research has shown that the oral–brain axis is a bidirectional communication system where oral health affects brain health and neurodegenerative alterations degrade oral function. This link is mediated by bacterial translocation, blood–brain barrier disturbance, systemic inflammation, and oral microbiome changes. *Porphyromonas gingivalis*, a periodontitis pathogen, has been found in Alzheimer’s brain tissue, and its virulence factors, gingipains, are linked to amyloid-β aggregation and tau protein hyperphosphorylation [14,15]. Due to this molecular commonality, oral health may be a modifiable neurodegenerative disease risk factor.

Despite possible biological explanations and observational evidence, the relevant literature is contradictory. This is because several works have utilized different evaluation techniques, populations, and oral and cognitive health standards [16]. The available material has been accumulated by systematic investigations on this topic, although discrepancies in approach and outcomes make conclusions problematic. An umbrella review, which entails reviewing earlier studies, is needed to synthesize and appraise evidence about dental health and cognitive loss in dementia due to this variation.

In the light of the growing body of evidence linking poor dental health to cognitive decline in individuals with dementia, this review seeks to synthesize and examine the findings of previously published narrative and systematic reviews, meta-analyses, and scoping reviews on the subject. By integrating current knowledge across multiple sources, this review aims to explore how oral health and Alzheimer’s disease may influence each other, with a focus on pathophysiological mechanisms, clinical associations, and potential preventive strategies.

## 2. Literature Review

A comprehensive narrative literature review was undertaken to examine the bidirectional relationship between oral health and Alzheimer’s disease. Literature searches were conducted in PubMed, Scopus, the Web of Science, and the Cochrane Library, targeting articles published between January 2010 and March 2024. The search strategy combined MeSH terms and free-text keywords, including concepts related to oral health (‘oral health’, ‘periodontal disease’, ‘tooth loss’, ‘oral microbiome’, ‘salivary biomarkers’, and ‘oral hygiene’) and cognitive impairment (Alzheimer’s disease’, ‘AD’, ‘cognitive decline’, and ‘neurodegeneration’). Filters were applied to include English-language studies on human subjects, as well as reviews and meta-analyses with full-text availability. Additional relevant publications were identified through manual screening of reference lists. The evidence was synthesized narratively and organized thematically, in accordance with the conceptual framework proposed by Popay et al. [17] The inclusion and exclusion criteria are detailed and centralized in Table 1:

✓ The inclusion criteria were (1) original studies (observational, cohort, case–control, or clinical), systematic reviews, or meta-analyses; (2) human participants, with or without AD; (3) a clear focus on the mutual influence between oral health and Alzheimer’s disease; and (4) publication in English, with full-text access.
✓ The exclusion criteria included (1) animal or in vitro studies; (2) articles not focused specifically on AD (e.g., general dementia); (3) editorials, letters, or abstracts without full text; and (4) non-English publications.

Following the selection process based on the predefined inclusion and exclusion criteria, the collected literature was examined in a narrative manner, aiming to capture the complexity and nuance of the association between oral health and Alzheimer’s disease. Rather than isolating individual findings, the focus was placed on identifying patterns, shared mechanisms, and areas of convergence across the existing reviews.

While the included studies varied in scope and methodology, most converged on the idea that the relationship is not unidirectional, but reciprocal; oral conditions may influence the course of Alzheimer’s disease, just as cognitive and functional decline can lead to deteriorating oral health. In order to explore this dynamic interplay, the synthesis was structured around these two interrelated perspectives.

### 2.1. Oral Health in Patients with Alzheimer’s Disease

A longer life expectancy and population aging have contributed to a marked rise in Alzheimer’s disease (AD), with its incidence estimated to have quadrupled globally over the past two decades [1,4]. AD typically manifests after the age of 65 and is associated with β-amyloid plaques and neurofibrillary tangles of hyperphosphorylated tau protein, leading to calcium dysregulation and cholinergic neuron loss [6,14,17]. More recent hypotheses suggest additional mechanisms, including oxidative stress and neuroinflammatory pathways [18]. These processes result in progressive cognitive and social decline, severely impacting quality of life in older adults.

**Figure 1:**
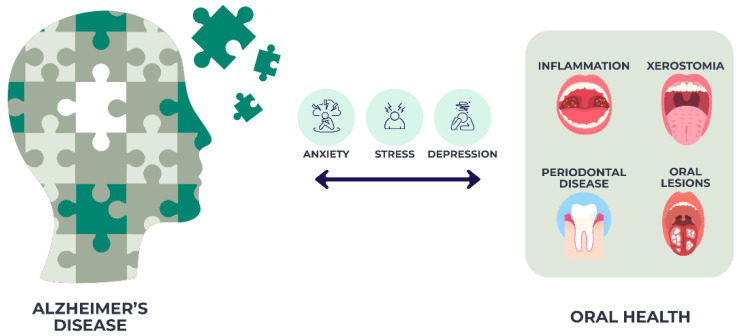
Oral health challenges in Alzheimer’s disease.

The relationship between Alzheimer’s disease (AD), dementia, and oral health has been widely studied. Older adults with AD generally present poorer dental health compared with those without dementia [19], a situation exacerbated by age-related motor and sensory decline that limits self-care and negatively affects oral health-related quality of life [20]. Dementia has been linked to higher prevalence of coronal and root caries, retained roots, and orofacial discomfort [21]. Periodontal problems, including bleeding gums, periodontitis, and attachment loss, are also more frequent in this population (Figure 1), along with xerostomia and oral lesions, such as stomatitis and candidiasis [22].

Mood stabilizers, benzodiazepines, antipsychotics, and other medications used as supplementary therapy or to alleviate cognitive symptoms in AD patients might worsen their oral health by causing serious adverse effects in the mouth [23,24]. Therefore, gerontology encompasses the maintenance of AD as an essential area. Alzheimer’s disease progressively affects oral health through declining cognitive and functional abilities, leading to poor oral hygiene, worsening dental and periodontal status, mucosal lesions, prosthetic difficulties, and altered salivary flow [25]. Together, these changes compromise oral function, comfort, and quality of life.

#### 2.1.1. Habits and Status of Oral Hygiene

Studies consistently show poorer oral hygiene in patients with Alzheimer’s disease (AD) and dementia compared with controls. Cestari et al. [26] found higher plaque index values in AD and MCI groups, while Chu et al. [27] reported that brushing twice daily was far less common in dementia patients (5% vs. 31%). Dementia patients also presented more visible plaque and greater difficulty with toothbrushing, although not all differences reached statistical significance [28].

#### 2.1.2. Dental Health and the Prevalence of Dental Cavities

Given the severe effects of tooth loss and untreated dental caries on systemic health, nutrition, and quality of life, geriatric dentistry and neurology are increasingly concerned with oral health in AD patients. The decayed, missing, and filled teeth (DMFT) index is used in clinical and epidemiological investigations to assess dental health and caries prevalence [28]. Several studies have linked elevated DMFT scores, AD, and tooth loss, suggesting a relationship between poor dental health and cognitive decline [28]. Two investigations demonstrated a substantial association between DMFT readings and AD diagnosis and increasing tooth loss [28,29]. This result was not universal.

Ribeiro et al. [30] reported mixed findings regarding oral health in AD patients. While one analysis showed no significant difference in DMFT scores between AD patients and cognitively healthy controls (*p* = 0.26), another demonstrated that AD patients had significantly higher DMFT values (*p* = 0.0002) and fewer natural teeth (*p* = 0.0004). These discrepancies may reflect methodological variability or the influence of confounding factors but overall suggest a greater burden of oral disease in the AD population.

Similarly, another study found that each missing tooth was predictive of moderate memory impairment (MMI) (*p* = 0.01) and that a lower tooth count was associated with increased likelihood of MMI (*p* < 0.05) [31]. Further supporting this trend, a separate investigation identified a positive correlation between the number of remaining teeth and MMSE scores in dementia patients [32], suggesting a potential impact of oral status on cognitive performance.

D’Alessandro et al. [33] also reported statistically significant differences in tooth decay (*p* = 0.005) and tooth loss due to reduced fillings in AD patients compared with healthy controls. Moreover, a large longitudinal cohort study that followed participants over a 10-year period found that a higher number of missing teeth was associated with an increased risk of developing dementia [34].

However, not all evidence aligns with these findings. For instance, Elsig et al. [35] reported that while dementia patients had more missing teeth than their controls, the difference was not statistically significant (*p* = 0.53). Furthermore, their study found no significant differences in DMFT components or in the number of sound teeth between the AD and control groups, contradicting other results in the literature [35].

Study design factors like AD severity, oral examination techniques, sample numbers, and dental care access may explain such inconsistencies. Even so, the evidence suggests that poor oral health, specifically tooth loss and high DMFT scores, is linked to cognitive impairment and dementia [36]. In a complete strategy to managing Alzheimer’s disease patients, early oral health assessment, preventive care, and regular dental monitoring are crucial. Table 2 summarizes significant studies on oral health markers such as DMFT scores and tooth loss and cognitive impairment in Alzheimer’s disease patients, showing both converging and inconsistencies.

#### 2.1.3. Periodontal Status

Studies show that periodontal inflammation and tissue degradation may be more common and severe in cognitively impaired Alzheimer’s disease (AD) patients. Periodontitis’ persistent inflammation and potential systemic consequences, including neuroinflammation, make this relationship important in AD development and progression [37].

AD patients have significantly higher levels for the gingival index (GI), periodontal index (PI), pocket probing depth (PPD), and clinical attachment loss (CAL) than cognitively healthy control groups [38]. Periodontal disease worsens when Alzheimer’s patients develop from mild to moderate to severe stages [37,38].

In a recent study, adverse events involving the alveolar bone process were more prevalent in the AD group (9.2%) compared with their controls (2.6%), with an odds ratio (OR) of 5.81, indicating a significantly increased risk of bone loss in dementia patients [38]. Additionally, a substantially greater proportion of teeth (56.2% vs. 17.1%) in AD patients exhibited deep periodontal pockets (≥6 mm), with the OR reaching 8.43 (95% CI: 1.14–29.68), suggesting a pronounced predisposition to advanced periodontal breakdown. The number of affected teeth in this group ranged between 4.00 and 17.76, further emphasizing the extent of periodontal involvement compared with the controls [38].

The findings from D’Alessandro et al. [33] reinforce this evidence, reporting significantly higher levels of clinical periodontal inflammation (CPI, *p* < 0.001) and gingival inflammation (GI, *p* < 0.001) among AD patients. These clinical parameters reflect the active disease status and may contribute to systemic inflammatory burden in individuals already predisposed to neurodegeneration [39].

Gao et al. [21] also highlighted that 64% of dementia patients presented with periodontal pockets, and 98% exhibited gingival bleeding, indicating a widespread presence of periodontal pathology in cognitively impaired individuals. However, their study did not find statistically significant differences in these periodontal characteristics—gingival bleeding, periodontal pockets, and attachment loss—between the dementia and non-dementia groups, possibly due to sample variability or population-specific confounders [21].

Similarly, a study by Chu et al. [27] did not observe a significant difference in the prevalence of periodontal pockets (CPI score ≥ 3) between the AD patients and controls (78% vs. 74%, *p* = 0.64). These findings were echoed in another investigation, which concluded that overall periodontal health did not differ significantly between the two groups, challenging the general assumption of a direct and uniform link between periodontitis and Alzheimer’s disease [40].

The levels of interleukin (IL)-6 were considerably higher in the AD patients compared with their controls (*p* = 0.029), while the levels of tumor necrosis factor-alpha (TNF-α) were markedly higher in periodontitis patients compared with AD patients with healthy periodontium (*p* = 0.005). PI (*p* = 0.008), PPD (*p* < 0.001), and CAL (*p* = 0.001), which are periodontal indicators, showed a positive correlation with TNF-α concentrations [26]. Serum TNF-α levels were greater in the patients with poor periodontal health. In the groups that were observed, there was a positive correlation between IL-6 and TNF-α (*p* < 0.001) [26].

Heterogeneity in study populations, clinical assessment methodologies, illness stage, and concomitant factors such dental hygiene, medication use, and systemic health issues may explain these inconsistent outcomes.

Cognitive decline may also worsen oral hygiene, thereby affecting periodontal disease in AD patients. However, most data suggests that Alzheimer’s patients have a larger burden of periodontal disease, especially moderate-to-severe cases. Periodontal infections and associated inflammatory mediators (e.g., IL-1β, TNF-α, and CRP) may cause systemic inflammation and worsen dementia, emphasizing the importance of early screening, prevention, and therapy in this population [41].

#### 2.1.4. Prosthodontic Status

The prosthodontic state of Alzheimer’s disease (AD) patients is crucial to their oral and general health. As cognitive decline develops, AD patients generally have trouble maintaining oral hygiene, resulting in tooth loss and prosthetic rehabilitation [42]. For functional restoration (e.g., mastication and speech), optimal nutrition, and quality of life, dental prostheses like complete dentures, partial dentures, and fixed prostheses must be evaluated [43]. Understanding prosthesis status in this population illuminates oral care needs and obstacles. Studies show that many AD patients are edentulous and may not use prosthetics or use ill-fitting or poorly maintained dentures, which can cause discomfort, mucosal lesions, impaired mastication, and social withdrawal [44]. Cognitive deficiencies can also reduce prosthetic appliance awareness and tolerance, resulting in low compliance or desertion [45].

According to research, 64.2% of AD patients and 58.3% of the non-AD group used dentures, with a *p* value of less than 0.05 [35]. Mild Alzheimer’s disease negatively impacts masticatory performance, according to case–control research by Campos et al. [46]. This impairment was seen in older adults with the condition, as measured by chewing efficacy and cognitive state. The chewing efficiency of dementia patients was shown to be lower than that of the controls, according to another study (*p* < 0.011) [35].

#### 2.1.5. Molecular Mechanisms Linking Periodontitis to Alzheimer’s Pathology

**Figure 2:**
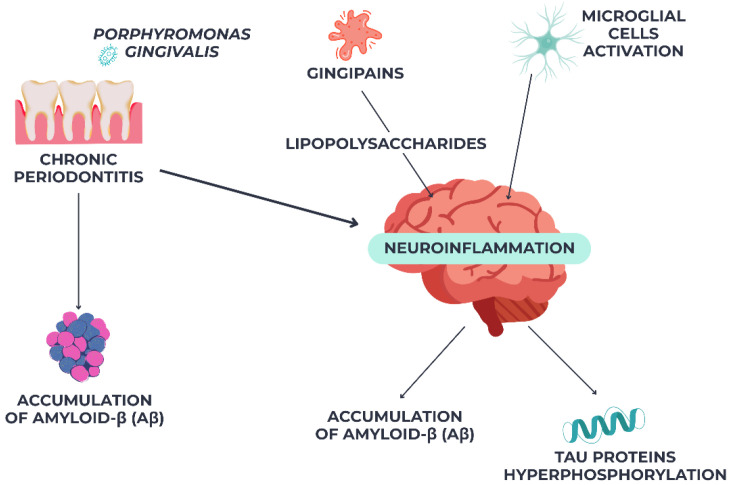
Pathogenic link between chronic periodontitis and Alzheimer’s disease via *Pophyromonas gingivalis*-mediated neuroinflammation.

Recent findings suggest biological mechanisms linking periodontal infections to neurodegeneration. *Porphyromonas gingivalis*, a periodontal infection, can cause amyloid-β (Aβ) buildup and tau protein hyperphosphorylation in the brain via translocation [47]. Gingipains, *P. gingivalis*’ proteolytic enzymes, have been found in Alzheimer’s patients’ brain tissue, suggesting a direct role in neuronal damage [48]. Periodontal lipopolysaccharides may also cause systemic inflammation, breach the blood–brain barrier (BBB), and activate microglial cells, causing chronic neuroinflammation and worsening Alzheimer’s pathology. As shown in Figure 2, these findings suggest a molecular link between chronic periodontitis and AD progression.

### 2.2. Influence of Oral Care in Patients with AD

Oral health is important yet often ignored in older Alzheimer’s disease (AD) patients. Maintaining good oral hygiene in elderly AD patients extends beyond tooth preservation. Poor dental health can lead to cardiovascular disease, diabetes, respiratory infections, and malnutrition, which this population is vulnerable to [49].

In addition, people with communication difficulties may not perceive oral pain and discomfort, causing behavioral symptoms including agitation, unwillingness to eat, or social isolation [50]. Advanced AD patients often require daily dental care from carers [51]. Carer training, time, and awareness of normal dental hygiene may be lacking [52]. Long-term care institutions may not prioritize individualized oral care, putting institutionalized patients at risk [53].

Malnutrition promotes poor dental health. Alzheimer’s patients often have poor dental health. Dental problems including tooth loss or xerostomia diminish sensory and masticatory capacities in the elderly, affecting nutritional intake [54].

Their growing oral disorders may cause oral and facial discomfort. Nonetheless, fewer people have experienced discomfort than expected. Although it is unclear if this is a natural result of Alzheimer’s disease, dementia, short-term memory loss, and other communication issues in the elderly may reduce discomfort. The elderly are distressed when pain, the body’s warning signal, lingers but is ignored [55].

Xerostomia, a dry mouth symptom, may be drug-induced or age-related. Several medications can cause xerostomia in Alzheimer’s patients [56]. Older Alzheimer’s patients have reduced stimulation and resting submandibular salivary flows. Poor dental health already lowers older individuals’ quality of life, and xerostomia worsens it. All of these factors make geriatric oral care crucial [57].

Alzheimer’s patients may have behavioral and cooperation issues when seeking dental care. Due to old age, mobility, systemic problems, and fragility, their therapeutic options may be limited. Due to impaired short-term memory and cognition, interacting with carers and receiving informed consent are vital [58,59]. After considering these considerations, prioritizing oral care can prevent oral illnesses and significant dental treatment [60]. This is possible when elderly Alzheimer’s patients receive high-quality dental care from their careers [61].

### 2.3. Biological Mechanisms Linking Oral Health and Alzheimer’s Disease

Dental health and Alzheimer’s disease are linked by microbiological, inflammatory, vascular, and neuronal pathways. Alzheimer’s disease can increase oral sickness, and several biological processes relate poor dental health to cognitive impairment and vice versa. These events may affect neurodegenerative pathways through blood–brain barrier disruption, microglial activation, and amyloidogenic protein aggregation [62]. However, as AD progresses, salivary function and oral hygiene can diminish, worsening oral disease and creating a vicious cycle [63]. This publication synthesizes current knowledge on the main biological pathways relating oral health to AD, incorporating novel molecular, experimental, and clinical results.

#### 2.3.1. Microbial Dissemination and Blood–Brain Barrier Disruption

**Figure 3:**
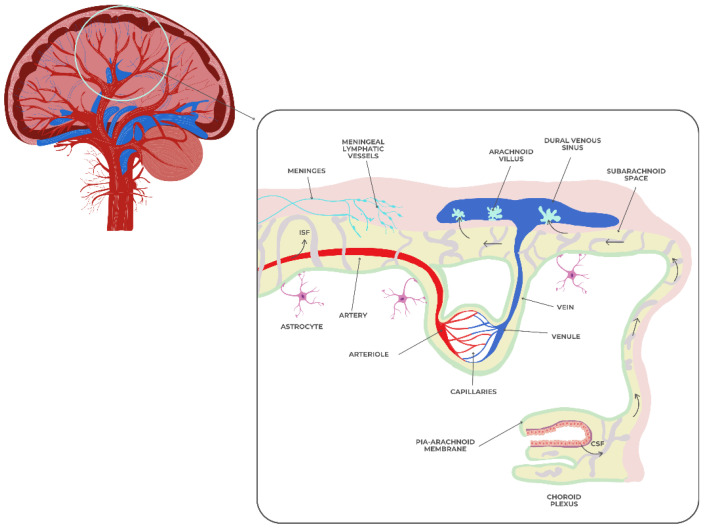
The blood–brain barrier.

The dynamic, selectively permeable blood–brain barrier (BBB) controls the molecular exchange between the central nervous system and systemic circulation. Figure 3 shows endothelial cells with tight connections, a basement membrane, pericytes, and astrocytic endfeet forming the BBB. These structures are linked to the meningeal layers, subarachnoid CSF, and cerebral vasculature [64]. Maintaining cerebral homeostasis and safeguarding the brain from poisons, infections, and peripheral inflammatory mediators requires BBB integrity.

*Porphyromonas gingivalis*, *Fusobacterium nucleatum*, and *Treponema denticola* can enter the bloodstream through ulcerated periodontal pockets, infected pulpal tissues, or chronic oral mucosal lesions [65,66]. Once in the bloodstream, these microbes or their virulence agents like lipopolysaccharides (LPSs) and gingipains can penetrate the BBB, especially if aging, hypertension, diabetes, or vascular pathology damage it [67].

Chronic oral infections increase systemic pro-inflammatory cytokines, such as IL-1β, IL-6, TNF-α, and CRP. These mediators can cross the blood–brain barrier and activate microglia, leading to neuroinflammatory responses that damage neurons, cause synaptic dysfunction, and lead to the buildup of Aβ plaques and hyperphosphorylated tau [68]. Poor dental health is associated with rapid cognitive decline due to sustained microglial activation, which drives neurodegeneration. DNA and antigens from oral infections were found in AD patients’ hippocampus and cortical regions after death, suggesting a causative role in neuropathology [64,67].

#### 2.3.2. Oral Microbiome Dysbiosis and the Oral–Gut–Brain Axis

A shift from symbiotic to pathogenic microbial communities in the mouth can affect brain function directly and indirectly. Swallowing oral infections affects gut flora and causes systemic endotoxemia. Inflammatory signals and dysbiosis-induced metabolites influence neuroinflammation via the oral–gut–brain axis [69].

Oral bacteria create amyloid-like proteins and proteolytic enzymes that may cause neurodegeneration. For instance, *P. gingivalis* gingipains degrade host proteins, hinder immunological modulation, and cause Aβ peptide aggregation [69]. Gipain inhibition reduced amyloid accumulation, neuroinflammation, and cognitive impairment in animal models, suggesting a dentistry-neurology therapeutic target [70].

Most SCFA-producing bacteria are colonic. Butyrate, acetate, and propionate are produced by these bacteria from dietary fibers. Gut health and brain activity depend on SCFAs [71]. Short-chain fatty acid (SCFA) levels in Alzheimer’s disease (AD) patients have changed, which may improve cognitive function [72].

To slow AD-related cognitive loss, SCFAs acetate, propionate, and butyrate are crucial. Clinical studies show that higher Aβ levels correlate with higher serum acetate and valerate levels, while butyrate levels are negatively related to Aβ levels [72]. SCFAs, produced by gut bacteria fermenting indigestible dietary fibers, inhibit neuroinflammation and microglial activation [73]. SCFAs, notably butyrate, improve cognition in Alzheimer’s disease by reducing peripheral inflammation and enhancing BBB integrity. They diminish neuroinflammation by activating microglia, which reduces cognitive deterioration [74].

SCFAs further increase cognitive performance by upregulating BDNF, a gene necessary for neurogenesis and synaptic plasticity. These effects, along with their BBB preservation and microbial balance, imply SCFAs may treat AD [72]. These findings also show how the gut microbiota affects the gut–brain axis, which explains how microbiome imbalances affect neurological illnesses like AD via inflammation, SCFA production, and blood–brain barrier integrity [75].

Many studies have examined the intricate relationship between the oral and gut microbiomes and their impact on health, but more is needed. For Alzheimer’s treatments to work, researchers must study microbial-host dynamics and gut-oral virome interactions. It’s intriguing to research how probiotics, prebiotics, vitamins, and diets may reduce systemic inflammation, microbial diversity, and immunological signaling.

### 2.4. Effective Strategies for Promoting and Preserving Oral Health in Patients with AD

Elderly dental health, especially in individuals with cognitive impairments like Alzheimer’s disease, requires a holistic and flexible approach. Prevention of oral diseases and quality of life when self-care declines require timely and concentrated therapies. Training patients and caretakers in preventative dental care is ideal. Training programs should emphasize daily fluoride toothpaste brushing and flossing (where applicable) and oral examination for inflammation, illness, or prosthesis concerns [76]. Use visual aids, shorter directions, electric toothbrushes, or new handles to promote compliance.

Regular dental care is essential for early oral disease detection and management. Dental examinations allow dentists to polish teeth and apply preventative treatments like fluoride varnish or sealants [77]. Dental professionals treating AD should use behavior management and see patients during their most cooperative times [78].

Institutional oral health guidelines are needed. Validated tools like the Oral Health Assessment Tool (OHAT) and standardized dental hygiene practices should be used regularly [79]. Overall care planning should incorporate oral health. Staff should prioritize helping uncomfortable, nonverbal individuals with oral health requirements.

Last but not least, dentists, doctors, nurses, nutritionists, and carers collaborate to maintain dental health. Working collaboratively, we can identify and treat oral health disorders such as medication-induced xerostomia [79,80].

Overall, individualized, consistent, and collaborative oral health treatments are essential, especially for at-risk older persons with cognitive loss. These programs improve oral health, communication, and psychological and social well-being.

**Figure 4:**
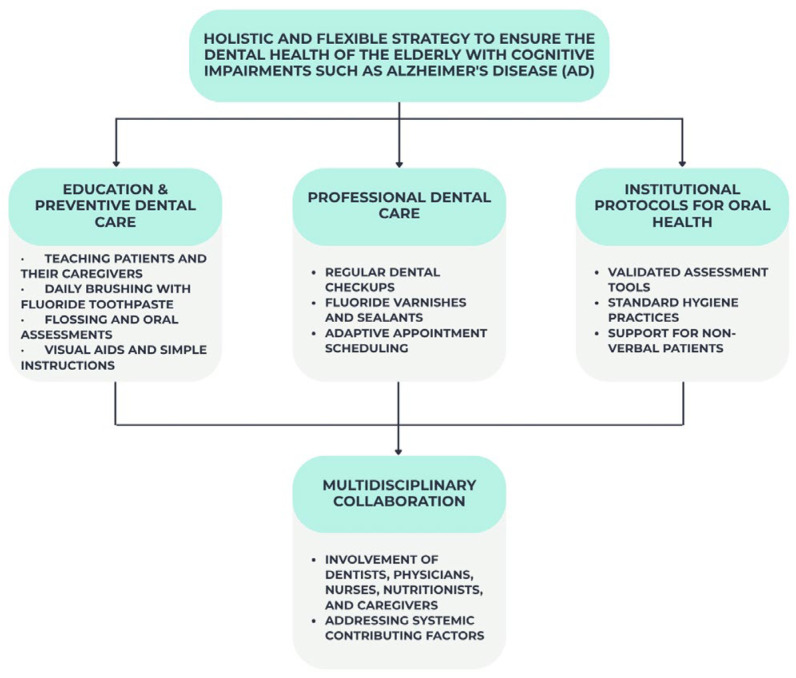
Key strategies for maintaining oral health in elderly patients with Alzheimer’s disease.

Figure 4 below summarizes the key interventions recommended for maintaining oral health in elderly individuals with Alzheimer’s disease, highlighting the need for a personalized, multidisciplinary approach tailored to the patient’s level of independence.

## 3. Future Perspectives

The urgent need to manage oral health in this susceptible demographic is growing in response to the aging population and the rising prevalence of Alzheimer’s disease. The oral health of Alzheimer’s disease patients, taking into account their individual cognitive and functional levels, should be the subject of future studies.

✓ Artificial Intelligence for Oral-Based AD Risk Screening

Artificial intelligence-powered smart toothbrushes, tele-dentistry tools, and tracking systems could change the way dental care is provided at home and in institutions by making it easier to brush and floss daily and finding dental problems early [81].

Recently developed technologies have made it easier for people who have trouble thinking and remembering to take better care of their teeth. According to a new study, ‘smart dentures’ with built-in sensors that measure things like bite force, temperature, and pH can give real-time information about how well the prosthesis is working and how well a person taking care of their teeth [82].

These kinds of improvements might help Alzheimer’s patients compensate for their declining ability to take care of themselves, making caregiving easier and raising the quality of life. To find out if these technologies are possible, useful, and effective in the clinic, as well as if they can be added to telemedicine platforms for ongoing, personalized monitoring of oral health, more research needs to be conducted in the future.

✓ Gut–Oral–Brain Axis: Emerging Mechanisms

The concept of a ‘gut–oral–brain axis’ highlights the complex interplay between oral dysbiosis, gut imbalance, and neurodegeneration. Oral pathogens such as *Porphyromonas gingivalis* can not only induce periodontitis but also alter the gut microbiome, increase intestinal permeability, and trigger systemic inflammation. The resulting cytokines (IL-1β, IL-6, and TNF-α) may cross the blood–brain barrier and accelerate neuronal damage, suggesting an indirect pathway through which periodontal disease impacts brain health [83]. Future studies should investigate this axis through longitudinal cohorts, microbiome-based biomarkers, and interventional strategies such as periodontal therapy and probiotics.

✓ Integrating Oral Health into Comprehensive, Personalized, and Multidisciplinary Dementia Care

The symbiotic link between dental health and cognitive deterioration also requires long-term research. Health outcomes for the elderly and those with dementia might be greatly improved by including dental care in regular management regimens [84]. Consistent and compassionate provision of dental care requires standardized and extended training programs for healthcare providers and carers.

Ultimately, a preventive, multidisciplinary, and person-centered approach will be essential for improving the quality of life of elderly individuals living with Alzheimer’s disease by preserving not only their oral health but also their dignity and comfort [84].

✓ Public Health Strategies and Policy for Oral Health Integration in Dementia Care

Given the bidirectional relationship between oral and cognitive health, there is a compelling need to integrate dental evaluation into routine geriatric assessments, particularly in patients with known cognitive decline. Public health strategies should prioritize access to preventive oral care in aging populations and promote caregiver education, especially in long-term care institutions.

Establishing interdisciplinary guidelines and protocols for oral care in dementia units, supported by trained dental professionals, could reduce disease burden and hospitalizations and improve quality of life. Furthermore, policies supporting routine screening for oral disease in older adults should be encouraged, including mobile dental units, domiciliary services, and tele-dentistry in underserved areas.

## 4. Conclusions

This review did not aim to provide definitive conclusions but rather to map and critically reflect upon the current body of evidence concerning the relationship between oral health and cognitive function in Alzheimer’s disease. By highlighting recurrent clinical themes—such as the associations between tooth loss, elevated DMFT scores, and cognitive impairment—while also acknowledging areas of conflicting results and methodological variability, the analysis underscores the complexity of this field and the limitations of existing studies.

One possible explanation for these clinical associations is the role of periodontal infection. Periodontitis, through its bacterial etiology—particularly pathogens such as *Porphyromonas gingivalis*—provides a biologically plausible pathway connecting oral disease with neurodegeneration. Gingipains and other virulence factors released by *P. gingivalis* have been shown to cross the blood–brain barrier, trigger microglial activation, and promote amyloid-β accumulation and tau hyperphosphorylation, mechanisms directly implicated in the onset and progression of Alzheimer’s disease. This strengthens the biological plausibility of the epidemiological associations identified in this review.

Ultimately, the synthesis of available evidence supports a paradigm shift in clinical practice: oral health should not be considered peripheral but a fundamental component of holistic dementia care. By integrating dental and neurological perspectives, management of Alzheimer’s disease can become more effective, equitable, and patient-centered.

Nevertheless, the evidence base remains fragmented, and the causal pathways are not fully elucidated. Therefore, there is an urgent need for well-designed, longitudinal, interdisciplinary studies to clarify these relationships and provide the foundation for evidence-based preventive and therapeutic strategies at the intersection of oral health and neurodegenerative disease.

## Figures and Tables

**Table 1:** Criteria applied to ensure relevance to the bidirectional relationship between oral health and Alzheimer’s disease.

Criterion	Inclusion Criteria	Exclusion Criteria
Study type	Original studies (observational, cohort, case–control, or clinical), systematic and narrative reviews, or meta-analyses	Animal or in vitro studies
Population	Human participants, with or without Alzheimer’s disease	Studies focused on general dementia without specific reference to AD
Focus of study	Clear examination of the mutual relationship between oral health and Alzheimer’s disease	Articles not addressing this relationship specifically
Language and access	Articles published in English, with full text available	non-English publications; abstracts, letters, editorials without full text

**Table 2:** Summary of studies linking oral health (DMFT index and tooth loss) with Alzheimer’s disease.

No.	Study and Author	Main Finding	Statistical Significance	Additional Notes
1	Okamoto et.al [28]	Each missing tooth predicted moderate memory impairment (MMI)	*p* = 0.01; *p* < 0.05	Association between tooth count and cognitive function
2	Ribeiro et al. [30]	Significantly higher DMFT scores in AD patients	*p* = 0.0002 (DMFT), *p* = 0.0004 (natural teeth)	Indicates greater oral disease burden in AD patients
3	Aragón et.al [31]	Strong correlation between DMFT values and AD diagnosis	Not specified	Supports link between poor oral health and AD
4	Budala et.al [32]	Positive correlation between remaining teeth and MMSE scores	Not specified	Suggests oral status impacts cognitive performance
5	D’Alessandro et al. [33]	Significant differences in decay and tooth loss due to fewer fillings in AD	*p* = 0.005	AD linked to fewer restorative treatments
6	Elsig et al. [35]	More missing teeth in dementia patients but not statistically significant	*p* = 0.53	No significant DMFT component differences found
7	Yoo et al. [36]	More missing teeth; increased dementia risk over 10 years	Not specified	Long-term follow-up study

## Abbreviations

The following abbreviations are used in this manuscript:

AD	Alzheimer’s disease
MeSH	medical subject headings
Aβ	amyloid-β
MCI	moderate cognitive impairment
PI	plaque index
DMFT	decayed, missing, and filled teeth
GI	gingival index
MMS	mini-mental state examination
IL-6	interleukin-6
OD	odds ratio
PPD	pocket probing depth
CAL	clinical attachment loss
CRP	protein C reactive
OHAT	Oral health Assessment Tool
SCFAs	short-chain fatty acids
